# Complete Genome Sequences for *Pseudomonas* sp. Strains 29A and 43A

**DOI:** 10.1128/MRA.00845-20

**Published:** 2020-09-24

**Authors:** David A. Baltrus, Meara Clark, Parris T. Humphrey, Noah K. Whiteman

**Affiliations:** a School of Plant Sciences, University of Arizona, Tucson, Arizona, USA; b School of Animal and Comparative Biomedical Sciences, University of Arizona, Tucson, Arizona, USA; c Department of Ecology and Evolutionary Biology, University of Arizona, Tucson, Arizona, USA; d Department of Organismic and Evolutionary Biology, Harvard University, Cambridge, Massachusetts, USA; e Department of Integrative Biology, University of California, Berkeley, California, USA; University of Southern California

## Abstract

*Pseudomonas* sp. strains 29A and 43A were originally isolated from the phyllosphere of individual plants of Cardamine cordifolia (Brassicaceae). Here, we report complete genome sequences for these two closely related strains, assembled using a hybrid approach combining Illumina paired-end reads and longer reads sequenced on an Oxford Nanopore MinION flow cell.

## ANNOUNCEMENT

Pseudomonads isolated from the environment are well known to produce a variety of molecules that either inhibit the growth of or actively kill other bacteria and fungi (1). Genome sequencing of environmental pseudomonads may therefore enable characterization and development of new therapeutic avenues for use across clinical and agricultural settings. To this end, we report the complete genome sequences for *Pseudomonas* sp. strains 29A and 43A, two closely related strains which display great potential as producers of antimicrobial compounds (2), originally isolated from the phyllosphere of Cardamine cordifolia (3).

Pure cultures of strains 29A and 43A were originally isolated from within surface-sterilized leaves from two individual plants by Humphrey et al. (3) and acquired by the Baltrus lab after a limited (<4) number of passages in rich medium on agar plates. These isolates were streaked to single colonies by the Baltrus lab onto King’s B (KB) agar plates. A single colony arising from these original platings was picked to 2 ml KB broth and grown overnight at 27°C, at which point a subset was frozen in −80°C in 40% glycerol to create stock cultures. For each genomic DNA extraction used in the assemblies reported here, a sample of this frozen stock was streaked onto KB agar plates; single colonies were transferred to 2 ml of KB broth and grown overnight at 27°C in a shaking incubator at 220 rpm. The genomic DNA used for Illumina sequencing was isolated from a 2-ml overnight culture via the Promega (Madison, WI) Wizard kit with the manufacturer’s protocols. The same genomic DNA isolation was used to create both Illumina and Nanopore libraries for strain 29A. Genomic DNA for Nanopore sequencing was prepared independently for each sequencing library for strain 43A, using the same methods described above. RNase was added as per the manufacturer’s protocols for all of the genomic isolations.

For each strain, DNA was sequenced by MiGS (Pittsburgh, PA) using an Illumina platform and following the standard workflow for library preparation and read trimming. As described by Baym et al. (4), this workflow uses an Illumina tagmentation kit for library generation, followed by sequencing on a NextSeq 550 instrument with 150-bp paired-end reads. Trimmomatic (5) was used for adapter trimming using the default settings. Genomic DNA from each strain (origin described above) was sequenced by the Baltrus lab via an Oxford Nanopore MinION instrument using an R9.4 flow cell, with 1 μg of DNA prepared using the LSK-109 kit without shearing or size selecting (other than using the long fragment buffer supplied with the kit). Reads were called during sequencing using Guppy version 3.2.6 using a MinIT (release 19.10.3) device for processing. Sequencing data for all read types are shown in Table 1.

**TABLE 1:** Sequencing data for *Pseudomonas* sp. strains 29A and 43A

Strain	Illumina data			Nanopore data			
	Total no. of reads	Genome size (bp)	Coverage (×)	Total no. of reads	*N*_50_ (bp)	Genome size (bp)	Coverage (×)
*Pseudomonas* sp. 29A	1,849,936	474,651,356	71	125,795	5,310	448,506,981	67
*Pseudomonas* sp. 43A	2,323,705	580,148,155	85	393,653	6,281	1,068,249,891	157

For each strain, hybrid assembly of all read types was performed using Unicycler version 0.4.8 (6). In each case, assembly resulted in a single chromosome, 6,775,260 bp with 59% GC content for strain 29A and 6,791,021 bp with 59% GC content for strain 43A. Both chromosomal replicons were determined to be circular, and both were rotated per the Unicycler pipeline. Chromosomes for both strains were annotated by the NCBI PGAP pipeline (7). Default parameters were used for all software.

### Data availability.

The genome project for strain 29A is indexed at GenBank and is associated with BioProject accession number PRJNA649619. The chromosomal sequence for strain 29A can be found under GenBank accession number CP059738.1. The Illumina reads can be found under SRA accession number SRX8856175. The FastQ files from the MinION run can be found under SRA accession number SRX8856176. The genome project for strain 43A is indexed at GenBank and is associated with BioProject accession number PRJNA638428. The chromosomal sequence for strain 43A can be found under GenBank accession number CP054880.1. The Illumina reads can be found under SRA accession number SRX8512942. The FastQ files from the MinION run can be found under SRA accession number SRX8512941. There is an additional set of Illumina reads for strain 43A available at SRX8512943, which were the product of a separate genomic DNA extraction, as described above. These were prepared and sequenced on a MiSeq instrument by SNPsaurus (Eugene, OR) but were not used in this reported assembly. Log files generated using Unicycler from both assemblies as well as a graph file (.GFA) resulting from both assemblies can be found at Figshare (https://doi.org/10.6084/m9.figshare.12585596).
